# Relative versus absolute rises in T/QRS ratio by ST analysis of fetal electrocardiograms in labour: A case-control pilot study

**DOI:** 10.1371/journal.pone.0214357

**Published:** 2019-03-26

**Authors:** Alexandra D. J. Hulsenboom, Kim M. J. Verdurmen, Rik Vullings, M. Beatrijs van der Hout–van der Jagt, Anneke Kwee, Judith O. E. H. van Laar, S. Guid Oei

**Affiliations:** 1 Department of Obstetrics and Gynaecology, Máxima Medical Centre, Veldhoven, the Netherlands; 2 Department of Electrical Engineering, Eindhoven University of Technology, Eindhoven, the Netherlands; 3 Department of Obstetrics and Gynaecology, University Medical Centre Utrecht, Utrecht, the Netherlands; Universidad de Zaragoza, SPAIN

## Abstract

**Introduction:**

The additional value of ST analysis during labour is uncertain. In ST analysis, a T/QRS baseline value is calculated from the fetal electrocardiogram and successive T/QRS ratios are compared to this baseline. However, variation in the orientation of the electrical heart axis between fetuses may yield different T/QRS baseline values. In case of a higher T/QRS baseline value more ST events are encountered, although not always related to perinatal outcome. We hypothesised that we can partly correct for this effect by analysing T/QRS rises as a percentage from baseline (relative ST analysis). This study aimed to explore whether relative ST analysis has better diagnostic value for cord acidaemia compared to conventional ST analysis, where predefined fixed T/QRS ratios are used.

**Methods and materials:**

A case-control study was performed in 20 term human fetuses during labour; 10 cases (umbilical cord artery pH <7.05 at birth, defining acidaemia) and 10 controls (pH >7.20) were included. The fetal electrocardiogram was recorded using a STAN monitor. We electronically extracted all T/QRS values, baseline and episodic ST events from the STAN monitor and calculated the relative T/QRS changes. The cut-off for relative ST events was determined in a receiver operator characteristic (ROC) curve at optimal specificity for cord acidaemia. Parameters of interest were area under the curve (AUC) of the ROC curve for relative ST events and test performance of both conventional and relative ST analysis.

**Results:**

Relative ST analysis showed an AUC of 0.99. The optimal cut-off value for relative T/QRS rise was determined at 0.70. Relative vs conventional (absolute) ST analysis showed a specificity of 100% vs 40% (p = 0.031); sensitivity 90% vs 90%; positive likelihood ratio infinity vs 1.5; negative likelihood ratio 0.10 vs 0.25, respectively.

**Conclusion:**

Relative ST analysis seems to be a promising method to detect impending fetal acidaemia during labour. Further studies are required to determine the diagnostic accuracy.

## Introduction

Fetal surveillance during labour, although performed continuously in labour wards around the world, is still a topic of debate. Due to poor specificity of cardiotocography (CTG), unnecessary caesarean deliveries are performed without improvement in long-term neonatal outcome [1]. ST analysis of the fetal electrocardiogram (ECG) (STAN, Neoventa Medical AB, Mölndal, Sweden) was introduced in the 1990s as a promising technique to accurately detect impending metabolic acidosis and improve perinatal outcome [2]. Metabolic acidosis can be objectively measured as the umbilical cord arterial pH and base deficit. Acidaemia in the umbilical cord is a marker of fetal distress during labour and is, most frequently, caused by hypoxia due to contractions. Two large randomised trials (RCTs), both comparing CTG monitoring alone to CTG monitoring plus ST analysis, showed promising results with a decrease in metabolic acidosis [3] and operative deliveries [2,3]. Subsequent RCTs did not confirm these results [4–7], and meta-analyses report both significant and non-significant decreases in metabolic acidosis [8–11].

The physiological rationale of ST analysis is based on the finding of Rosén et al. [12–14] that hypoxia in fetal lambs leads to an adrenalin surge, resulting in local glycogenolysis and potassium release in the fetal myocardium. This local increase in potassium ions leads to an increase in T wave amplitude in the fetal ECG [12,14,15]. In ST analysis this T wave amplitude is quantified as the ratio between the T wave amplitude and the QRS amplitude, the so-called T/QRS ratio. At the start of the fetal ECG registration, a T/QRS baseline is determined in a 2-step procedure. First, for every T/QRS ratio a median is calculated over the last 20 preceding T/QRS ratios. Then, the T/QRS baseline is defined as the lowest value of these median T/QRS ratios within a three hour window preceding the current T/QRS ratio. At the same time, for every T/QRS ratio another median is calculated over the last 10 preceding T/QRS ratios, from here on defined as T/QRS-med10. In case there is a rise in T/QRS ratio, defined as a difference between T/QRS ratios and the baseline, that exceeds a predefined threshold, an alarm is automatically triggered and reported by the STAN monitor. ST analysis discriminates three types of events (alarms): episodic, baseline, and biphasic events. In case T/QRS-med10 exceeds the baseline by 0.05 –analogous to a rise of T/QRS ratios above the baseline for at least 10 minutes—a baseline ST event is reported [15]. When the current T/QRS ratio exceeds the T/QRS-med10 by 0.10 –analogous to a rise of T/QRS that lasts shorter than 10 minutes—an episodic ST event is reported. Both episodic and baseline events represent an absolute increment of the T/QRS ratio compared to the T/QRS baseline. Biphasic ST events are defined as ST intervals with a downward slope [15]. The relevance of a ST event depends on the visual assessment of the CTG. In case the CTG is classified as normal, all ST events given by the STAN monitor can be ignored [16–18]. In that case, the ST event is classified as a non-significant event. In clinical practice, such non-significant events are frequently encountered [19]. In case the CTG is classified as intermediary or abnormal, depending on the classification of the CTG trace, ST events might be considered to be significant and a clinical intervention should be prompted [18]. The dependence on subjective CTG interpretation, which is known to have a large inter- and intra-observer variability [1,20], is the Achilles’ heel of CTG plus ST analysis in clinical practice.

Previously, we described a physiological explanation for false positive ST events and hypothesised that relative ST analysis (T/QRS rises as a percentage from baseline) could reduce these false events. We demonstrated that the orientation of the fetal electrical heart axis varies considerably between fetuses [21]. The relation between the orientation of the electrical heart axis and the orientation of the scalp electrode yields different T/QRS baseline values, due to differences in ECG morphology. We found that fetuses with a higher T/QRS baseline are more prone to ST events, independent of the fetal condition [22]. In contrast, we found that fetuses with a lower T/QRS baseline are less prone to exceed the threshold for a ST event. In other words, it is expected that a high initial T/QRS baseline increases the incidence of false positive ST events and that a low initial T/QRS baseline increases the incidence of false negative ST events. Besides, Becker et al. [23] described that higher T/QRS baseline values are not related to poor neonatal outcome or hypoxia.

Both the T/QRS baseline value and the rise in T wave amplitude are affected by the orientation of the electrical heart axis, although to different extents. The amplitude of the QRS complex and amplitude of the T wave in the ECG reflect the amplitude of the electrical activity of the heart in the direction of the scalp lead. The ECG amplitude depends on both the amount of electrical activity and the orientation of this activity, i.e. the geometrical angle between the direction of the electrical activity and the direction of the scalp lead. Because the electrical activity of the QRS complex and the T wave have different orientations with regard to the scalp lead, variations in the orientation of the electrical heart axis affect the QRS complex and the T wave to different extents, hence affecting the T/QRS ratio.

As mentioned above, we hypothesised that analysing relative, rather than the conventional, ‘absolute’ T/QRS rises from baseline, could improve the diagnostic value of ST analysis. In case of relative ST analysis (RSTAN) (T/QRS rises as a percentage from baseline), there is a correction for the ‘ease’ of increment of the T wave amplitude. In conventional absolute ST analysis (ASTAN), an absolute rise in T/QRS value will lead to an event when above a certain predefined fixed increase, irrespective of the height of the T/QRS baseline. This study aimed to explore the prospect whether RSTAN could have a diagnostic value in detecting impending cord acidaemia. In addition, we aimed to compare sensitivity, specificity, positive and negative likelihood ratios of both RSTAN and ASTAN. This is the first study that describes RSTAN. In order to directly compare both methods, we only focused on objective information. Therefore, we omitted subjective CTG interpretations.

## Materials and methods

### Patient inclusion

We performed a case-control study, using the complete dataset previously collected by van Laar et al. [24]. The ethics committee of Máxima Medical Centre decided that there was no need to acquire written informed consent from the included patients given the retrospective nature of this study. The criteria for inclusion were equal to those in the original dataset [24]. We included fetuses of at least 36 weeks of gestation with intrapartum fetal ECG recordings, whose arterial and venous umbilical cord blood gases were determined directly after birth. The included mothers were healthy, had an uncomplicated pregnancy and did not use any medication except oxytocin or epidural analgesia during labour. We only included fetuses with a normal 20 week anomaly scan including echocardiography. Only good quality fetal ECG recordings were included, defined as absence of ectopic beats and no missing data in the last 10 minutes before birth. We excluded fetuses with fetal growth restriction (defined as birth weight below the 10^th^ percentile), because these fetuses might have myocardial hypertrophy with, hypothetically, a deviated heart axis [25–27]. For all 20 fetuses, good quality ECG data were available until nine minutes before birth. In nine fetuses (four cases, five controls), fetal ECG data could be obtained until the last minute before birth. The median duration of the recordings was 277 minutes, the duration ranged from 42 to 524 minutes.

Cases had an umbilical artery cord pH <7.05 and controls had an umbilical artery cord pH >7.20. We chose the cut off at pH >7.20 for the controls as it represents the upper range of normality and represents the group of uncompromised fetuses. Cases were consecutively selected between January 2006 and December 2007 in the Máxima Medical Centre, Veldhoven, the Netherlands. As a result of the strict inclusion and exclusion criteria, only five fetuses with acidaemia could be included. Therefore, five additional fetuses with acidaemia were consecutively selected from the University Medical Centre Utrecht, the Netherlands, between January 2001 and July 2002. Both hospitals are tertiary-care teaching hospitals. The ten controls were consecutively selected between January 2007 and August 2007 in the Máxima Medical Centre, Veldhoven, the Netherlands.

### Signal processing

**Fig 1:**
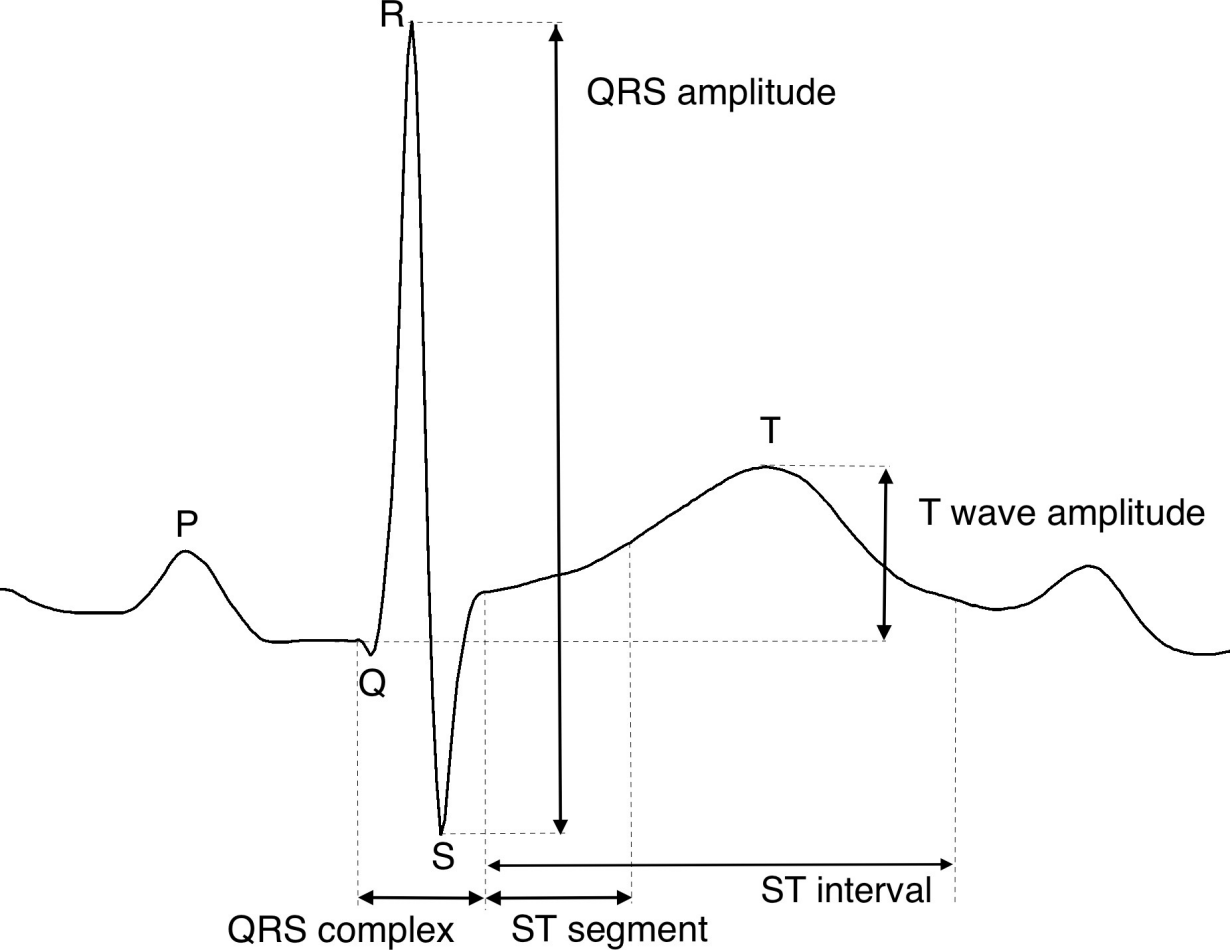
Schematic ECG parameters. The T/QRS ratio is the quotient of T amplitude and QRS amplitude.

Fetal ECG recordings were obtained during labour with a single helix scalp electrode (Goldtrace), a maternal skin electrode, and STAN S21 or S31 monitors (Neoventa Medical AB, Mölndal, Sweden). The STAN monitor detected an ECG complex for every heartbeat. After 30 good quality ECG complexes, an average ECG complex was automatically calculated. This average ECG complex was used to determine the amplitudes of the T wave and QRS complex which, in turn, were used to calculate a single T/QRS ratio after every 30 heart beats (Fig 1). We extracted all these T/QRS ratios from all registrations.

The STAN monitor automatically reports episodic, baseline and biphasic events in an ‘event log’. We used this ‘event log’ in the STAN viewer software (Neoventa) to determine whether baseline or episodic ST events occurred. Biphasic events were not evaluated in this study, since only baseline dependent ST events are known to be related to the orientation of the electrical heart axis [22]. In addition, the value of biphasic events is under debate, as recent studies showed they do not discriminate in the prediction of fetal distress or adverse outcome [19, 28, 29].

For RSTAN, instead of assessing the difference between T/QRS ratios and the baseline, we calculated the quotient. We defined the baseline equally to the STAN method as described in the Introduction. In case the 20 consecutive preceding T/QRS ratios did not fall within a 20-minute window from the current T/QRS ratio, we classified the signal as low quality and did not update the baseline (the baseline remained unchanged; this method is similar as in the STAN monitor). All calculations were performed electronically in Matlab (The Mathworks, Natick, MA). To determine the optimal threshold for RSTAN, the largest T/QRS rise from baseline per patient was plotted in a receiver operating characteristic curve (ROC curve) in SPSS 22 for Mac (IBM corp. Armonk, NY, USA). The ROC curve was calculated under non-parametric assumption. The cut-off points for which sensitivity and specificity were calculated were chosen by SPSS. The smallest cut-off value was the minimum observed relative T/QRS rise minus 1, and the largest cut-off value is the maximum observed relative T/QRS rise plus 1. The other cut-off values were the averages of two consecutive ordered observed relative T/QRS rises. The optimal threshold to predict umbilical cord arterial acidaemia was determined from the ROC curve (the point closest to (0,1)).

As mentioned previously, we wanted to assess the test performance of ASTAN and RSTAN in a cohort of 10 cases and 10 controls. Ideally, alarms should appear in the pH < 7.05 group (cases). On the other hand, we would expect no alarms in the pH > 7.20 group (controls). We considered a registration to have at least one relative ST event, in case the largest relative T/QRS rise in a registration exceeded the previously defined threshold for RSTAN. To assess whether there was an absolute ST event, we checked whether the event log of the STAN monitor reported any baseline or episodic ST event. We omitted biphasic ST events. In addition, we reported whether there where any absolute or relative ST events in the last 42 minutes of the registration, which was the duration of the shortest registration. To omit subjective interpretation of the CTG, no CTG classification was performed. Therefore, absolute ST events were not classified as significant or non-significant. Instead, we classified the events as true or false, based on whether the patient was a case or a control.

### Statistical analysis

Patient characteristics were listed in Table 1. Categorical variables were presented as frequency and percentage. Continuous variables were presented both as mean and standard deviation, and median and total range. We reported the area under the receiver operating characteristic (AUC) for RSTAN and the p value (compared to the null hypothesis of an AUC of 0.5). We used a ROC curve to determine a threshold for RSTAN at the point closest to (0,1) and at optimal specificity. This threshold was subsequently used to compute positive and negative likelihood ratios of RSTAN. RSTAN and ASTAN were compared with respect to sensitivity, specificity, positive likelihood ratio (LR+) and negative likelihood ratio (LR-) to detect impending umbilical cord acidaemia (pH <7.05). We used a McNemar test to compare RSTAN to umbilical cord arterial acidaemia, ASTAN to umbilical cord arterial acidaemia, specificity between RSTAN and ASTAN and sensitivity between ASTAN and RSTAN. A p-value <0.05 was considered to be statistically significant. In addition, we tested agreement between RSTAN and ASTAN with Cohen’s unweighted Kappa and the composite proportions of agreement with VassarStats online software (Lowry, Avon, USA Available from: http://vassarstats.net).

**Table 1:** Patient characteristics.

Characteristic	Cases (n = 10)		Controls (n = 10)	
Nulliparous–n (%)	8 (80)		4 (40)	
Gestational age–days	283 ±8	283 [269–295]	278 ±11	279 [259–294]
Epidural analgesia–n (%)	3 (30)		3 (30)	
Oxytocin–n (%)	7 (70)		4 (40)	
FSBS–frequency	1.4	2 [0–3]	0	0
Birth weight–gram	3414 ±423	3375 [2910–4110]	3643 ±562	3690 [2770–4500]
Arterial pH	6.98 ±0.07	7 [6.82–7.04]	7.26 ±0.03	7.26 [7.20–7.29]
Arterial Base Excess–mmol/l	-17.3 ±4.0	-17 [11–26]	-5.2 ±2.0	-5.5 [2.0–9.0]
Venous pH	7.08 ±0.10	7.09 [6.87–7.19]	7.33 ±0.04	7.34 [7.25–7.39]
Hospital admission–n (%)	4 (40)		0 (0)	
Ventouse delivery* –n (%)	4 (40)		0 (0)	

Abbreviations: FSBS = fetal scalp blood sampling. Continuous variables are presented as both mean ± standard deviation and median [total range minimum-maximum].

*None of the included patients had a cesarean or forceps delivery

## Results

### Patient characteristics

Table 1 shows the patient characteristics. None of the included patients had a cesarean section or forceps delivery. Four cases had a ventouse delivery; no operative deliveries were performed in the control group.

### Relative versus absolute ST analysis

**Fig 2:**
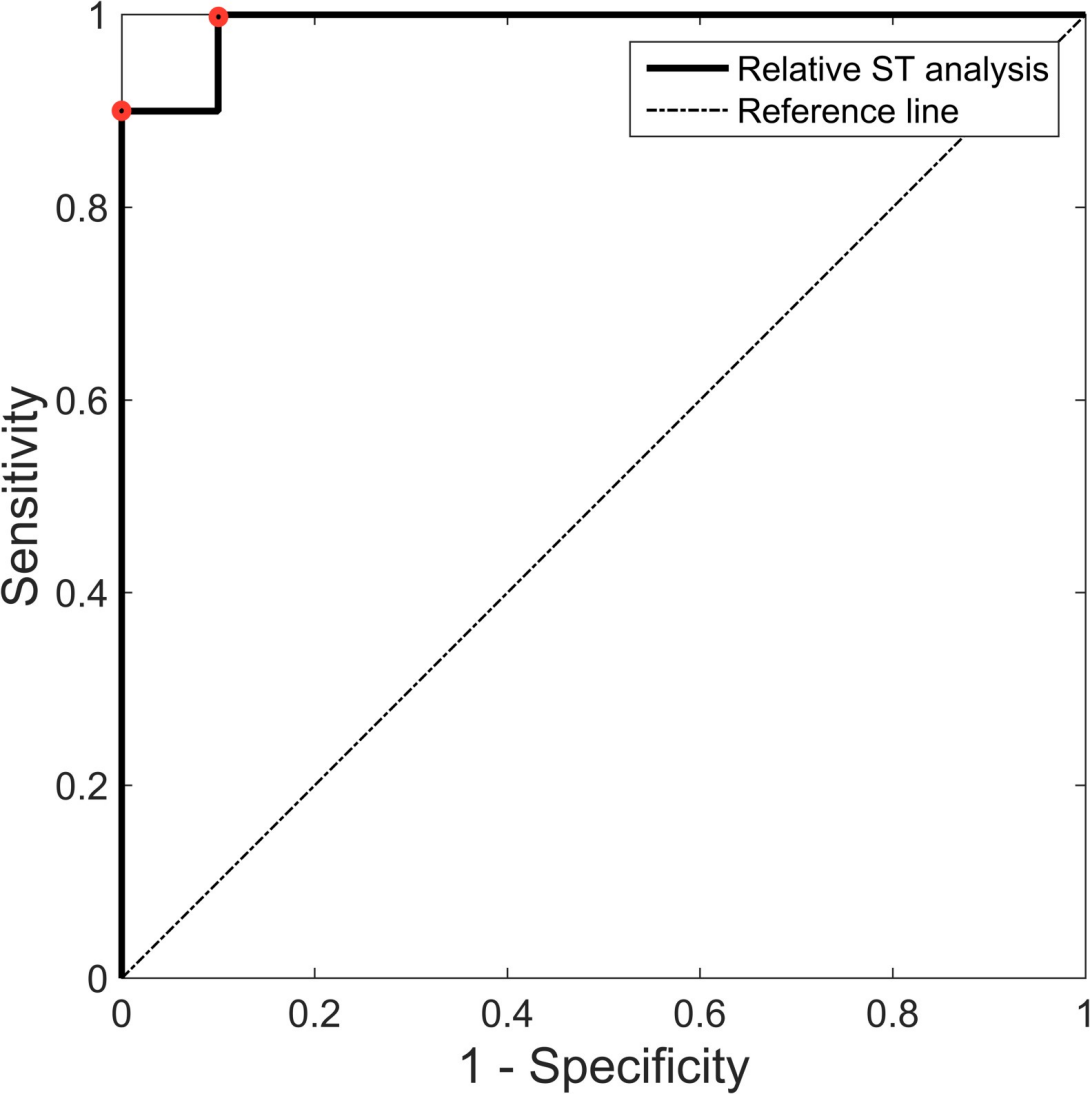
Receiver operating characteristic (ROC) curve of relative ST analysis. The ROC curve was calculated under non-parametric assumption. The two highlighted points (red dot) represent the cut-offs closest to (0,1) at a relative T/QRS ratio rise of 0.64 (sensitivity 100%, specificity 90%) and 0.70 respectively (sensitivity 90%, specificity 100%).

Fig 2 shows the ROC curve for RSTAN. The area under the ROC curve for relative T/QRS ratio changes in this population was 0.99 (p <0.001). Two optimal cut-off values were determined, at a relative T/QRS ratio rise of 0.64 (specificity 90%, sensitivity 100%) and 0.70 (specificity 100%, sensitivity 90%), respectively. Both cut-off values were equally close to (0,1). We chose to use 0.70 as cut-off for the subsequent analyses (i.e. a 70% rise of the T/QRS ratio from baseline), as it had better specificity and poor specificity is the main problem of the available monitoring techniques.

**Fig 3:**
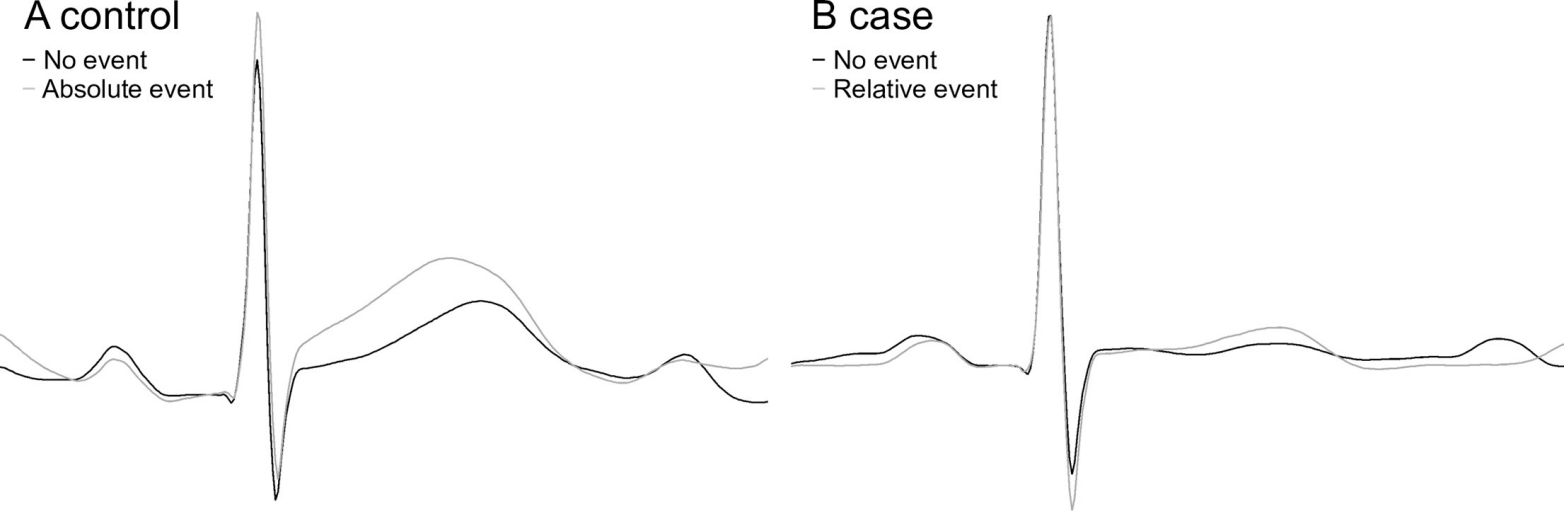
ECG examples. Panel A shows an example of an ECG in a control (pH>7.20) at the beginning of the registration (black) and during an absolute (episodic) ST event (grey). The absolute rise in T/QRS ratio here exceeds 0.10, whereas the relative T/QRS rise does not exceed 0.70 (no event). Panel B shows an ECG in a case (pH<7.05) at the beginning of the registration (black) and during a relative ST event (relative rise from baseline is 1.25) (grey). The absolute T/QRS rise is here below 0.05 (no absolute ST event).

Fig 3 shows an example of an ECG at the beginning of the registration and during an event in a control (A) and a case (B) respectively. It illustrates that for a patient with pH>7.20 a change in T/QRS ratio can occur that is classified as an absolute ST event without being a relative ST event (control). Vice versa, for a patient with pH<7.05, a change in T/QRS ratio can occur that is not classified as a absolute ST event, but that is classified as a relative ST event (case).

We compared the test performance of ASTAN and RSTAN in our study population (Tables 2 and 3). Sensitivity was equal for both methods, while specificity, LR+, and LR- seemed to be better for RSTAN.

**Table 2:** Crosstable of relative and absolute ST analysis.

		Case: pH <7.05 (n = 10)	Control: pH >7.20 (n = 10)
**Relative ST analysis**	Event	9	0
	No event	1	10
**Absolute ST analysis**	Episodic or baseline event	9	6
	No event	1	4

Relative ST analysis was defined as at least one relative T/QRS rise from baseline over 70%. Absolute ST analysis was defined as at least one reported baseline or episodic event in the event log. None of the cases had any episodic event. 4 controls had solely baseline events, 1 control had at solely episodic events, and 1 control had both baseline and episodic events.

**Table 3:** Test performance of absolute and relative ST analysis to detect impending cord acidaemia (pH < 7.05).

	Absolute ST analysis	Relative ST analysis
**True positive—**n/total	9/10	9/10
**True negative—**n/total	4/10	10/10
**Sensitivity**	0.9	0.9
**Specificity**	0.4	1.0
**Positive likelihood ratio**	1.5	∞
**Negative likelihood ratio**	0.25	0.1

Absolute ST analysis was defined as at least one reported baseline or episodic event in the event log. Relative ST analysis was defined as at least one relative T/QRS rise over 70% from baseline. Test performance was depicted as sensitivity (true positives/(true positives + false negatives)), specificity (true negatives/(true negatives + false positives)) positive likelihood ratio (sensitivity/(1—specificity)) and negative likelihood ratio ((1 –sensitivity)/specificity). Positive outcome was defined as cord artery acidaemia (pH <7.05).

In addition, we compared the presence of absolute and relative ST events in the last 42 minutes of each registration (same registration length). ASTAN and RSTAN showed a sensitivity of respectively 70% and 60% and specificity of respectively 60% and 100%.

When using a cut-off value of 0.70 as threshold for RSTAN, RSTAN and ASTAN had comparable sensitivity (McNemar p = 1.000), but differed significantly regarding specificity (McNemar p = 0.031) to discriminate acidaemic and non-acidaemic fetuses. Overall, RSTAN agreed well with cord acidaemia (McNemar p = 1.000), while ASTAN tended to agree less well (McNemar p = 0.125). RSTAN and ASTAN showed a weak agreement (Cohen’s unweighted kappa 0.42). The composite proportion of agreement between RSTAN and ASTAN was moderate (0.72).

## Discussion

This study explored the possibility whether RSTAN in comparison with ASTAN could have a better diagnostic accuracy in detecting umbilical cord arterial acidaemia, while restricting the number of false positive events. We found that RSTAN may improve the identification of fetuses with impending acidaemia at birth. To explore whether RSTAN could solve the frequently occurring problem of non-significant ST events with ASTAN [19], we compared the sensitivity and specificity of both methods. In this pilot study, in which 50% of the included patients had cord blood acidaemia, RSTAN indicated to have a better specificity, and a trend towards better positive and negative likelihood ratios. Sensitivity was equal. The agreement between ASTAN and RSTAN was weak to moderate, indicating that RSTAN might be better in identifying impending cord acidaemia during labour.

In ASTAN, non-significant ST events are frequently encountered [20]. This could lead to alarm fatigue of the staff, which might have deleterious consequences for both the mother and child. This study indicated that ST monitoring with correction for orientation of the electrical heart axis may be capable of identifying fetuses with acidaemia without showing many false positive events. However, as CTG interpretation was not included in the study, it is unknown whether ASTAN events would have been classified as truly significant or non-significant. We suggest that as a topic for further studies.

In case comparing the last 42 minutes of each registration, sensitivity of RSTAN was 60%, while it was 90% in the total registration. This difference can be explained by the fact that a shorter registration misses earlier RSTAN events.

Lee et al. described that the electrical heart axis may change during tachycardia and decelerations [30]. We are aware that this could introduce a bias in both ASTAN and RSTAN methods. However, we expect this error to be smaller in RSTAN, as this method corrects for variations of the electrical heart axis.

Although the results from this first study describing RSTAN are promising, there are some important limitations. We did not evaluate the full spectrum of patients in a clinical setting, since only cases with proven acidaemia and controls with normal umbilical cord pH values at birth were included. A large middle group with umbilical cord pH between 7.05 and 7.20 was not represented, Furthermore, the study sample size was small; only 10 cases and 10 controls, which makes the risk of both type I and type II errors high.

Taken the limitations of our study into account, validity of RSTAN should be evaluated in a larger patient group, including the full spectrum of perinatal outcomes. This would allow to determine a more reliable and representative threshold value for RSTAN. Subsequently, such a threshold should then be validated in a different group of patients. We then expect that the test characteristics of RSTAN will be less optimistic than those found in the current work. Unfortunately, there is a lack of large datasets with high quality fetal ECG data stored electronically, accompanied by complete and relevant clinical information. This delays further progress in this field of research.

## Conclusion

This study indicates that relative T/QRS analysis is a promising technique to detect impending fetal acidaemia during labour. In comparison to conventional absolute ST analysis, relative ST analysis might have better specificity with a comparable sensitivity. Relative ST analysis seems to be a promising method for monitoring of fetal wellbeing during labour and needs to be studied in a larger population.
